# Dose- and time-dependence of the host-mediated response to paclitaxel therapy: a mathematical modeling approach

**DOI:** 10.18632/oncotarget.23514

**Published:** 2017-12-20

**Authors:** Madeleine Benguigui, Dror Alishekevitz, Michael Timaner, Dvir Shechter, Ziv Raviv, Sebastien Benzekry, Yuval Shaked

**Affiliations:** ^1^ Cell Biology and Cancer Science, Rappaport Faculty of Medicine, Technion, Israel Institute of Technology, Haifa, Israel; ^2^ MONC Team, Inria Bordeaux Sud-Ouest and Institut de Mathématiques de Bordeaux, Talence, France

**Keywords:** chemotherapy, host effects, mathematical models, invasion and migration, metronomic chemotherapy

## Abstract

It has recently been suggested that pro-tumorigenic host-mediated processes induced in response to chemotherapy counteract the anti-tumor activity of therapy, and thereby decrease net therapeutic outcome. Here we use experimental data to formulate a mathematical model describing the host response to different doses of paclitaxel (PTX) chemotherapy as well as the duration of the response. Three previously described host-mediated effects are used as readouts for the host response to therapy. These include the levels of circulating endothelial progenitor cells in peripheral blood and the effect of plasma derived from PTX-treated mice on migratory and invasive properties of tumor cells *in vitro*. A first set of mathematical models, based on basic principles of pharmacokinetics/pharmacodynamics, did not appropriately describe the dose-dependence and duration of the host response regarding the effects on invasion. We therefore provide an alternative mathematical model with a dose-dependent threshold, instead of a concentration-dependent one, that describes better the data. This model is integrated into a global model defining all three host-mediated effects. It not only precisely describes the data, but also correctly predicts host-mediated effects at different doses as well as the duration of the host response. This mathematical model may serve as a tool to predict the host response to chemotherapy in cancer patients, and therefore may be used to design chemotherapy regimens with improved therapeutic outcome by minimizing host mediated effects.

## INTRODUCTION

One of the major obstacles in clinical oncology is that tumors acquired resistance to therapy and relapse, despite an initial response to therapy. While the main reasons for tumor relapse are related to intrinsic and acquired resistance mechanisms within tumor cells, a growing body of literature suggests that host-mediated effects generated in response to therapy may counteract the anti-tumor activity of the drug, thereby contributing to decreased net therapeutic outcome. The host response to therapy is composed of both molecular and cellular changes in peripheral blood circulation [1]. We and others have identified several soluble factors induced in response to therapy that contribute to tumor regrowth and metastasis. These factors include metalloproteinase 9 (MMP9), IL16, IL1β, SDF-1, Osteopontin, stem cell factor, and granulocyte colony stimulating factor among others [2]. This storm of cytokines and growth factors is followed by acute mobilization of cells from the bone marrow compartment and their homing to the tumor site, including endothelial progenitor cells [3], Tie-2 expressing monocyties [4], mesenchymal stem cells [5], myeloid derived suppressor cells [6] and tumor associated macrophages [7]. Together, the response of the host to anti-cancer drug therapy contributes to pro-tumorigenic and pro-metastatic activities by promoting angiogenesis and inhibiting immunity against tumor cells.

The administration of chemotherapy drugs contributes to tumor cell aggressiveness by different means. For example, it has been shown that tumor cells cultured in the presence of plasma obtained from naïve mice 24 hours after treatment with the maximum tolerated dose (MTD) of paclitaxel (PTX) or gemcitabine chemotherapy exhibit increased migratory and invasive potential [8]. In addition, treating tumor-bearing mice with cytotoxic agents such as vascular disrupting agents or PTX chemotherapy induces a rapid mobilization of bone marrow derived circulating endothelial progenitor cells (CEPs) that home to the treated tumor site and facilitate angiogenesis ultimately leading to tumor cell repopulation and re-growth [4, 9]. Indeed, several studies have indicated that while cytotoxic anti-cancer drugs induce tumor cell killing right after drug administration, during the drug-free break periods in between successive MTD treatments, tumor cells proliferate and repopulate the site [10, 11]. This could be explained by the systemic host-mediated effects generated in response to the acute administration of chemotherapy that induces a variety of pro-angiogenic and pro-metastatic mechanisms. In contrast, such host-mediated effects are minimized with the use of low-dose metronomic chemotherapy (LDM), which is administered more frequently, and with no extended drug-free break periods [12, 13]. This may explain, at least in part, the therapeutic benefit reported for LDM [14, 15]. However, to date, the host response to different dosages and duration of chemotherapy has not been fully characterized.

Quantitative mathematical methods have recently emerged in oncology as powerful tools to test mechanistic hypotheses against experimental observations and predict the effects of varying the scheduling of anti-cancer drug regimens [16]. Recent examples include theories and methods of prediction for experimental tumor growth [17, 18] or pre- and post-surgical metastasis [19], as well as mathematical models for the rational design of metronomic schedules [20, 21] and combination of anti-angiogenics and cytotoxic drugs [22].

Here we studied the complexity and dynamics of the host response, focusing solely on the effect of the drug on the host. To do so, we used non-tumor bearing mice treated with PTX chemotherapy. This approach allowed us to distinguish between the anti-tumor activity of the drug and the pro-tumorigenic activity generated by the host in response to the therapy. We used a mathematical modeling approach to predict the host responses to PTX chemotherapy. It is based on the dose of the drug and the duration of the host response after drug administration. Our results demonstrate a model that accurately predicts host-mediated responses to chemotherapy. Therefore, it may serve as a tool to design chemotherapy regimens with improved therapeutic outcome.

## RESULTS

### Differential host response to PTX chemotherapy

**Figure 1:**
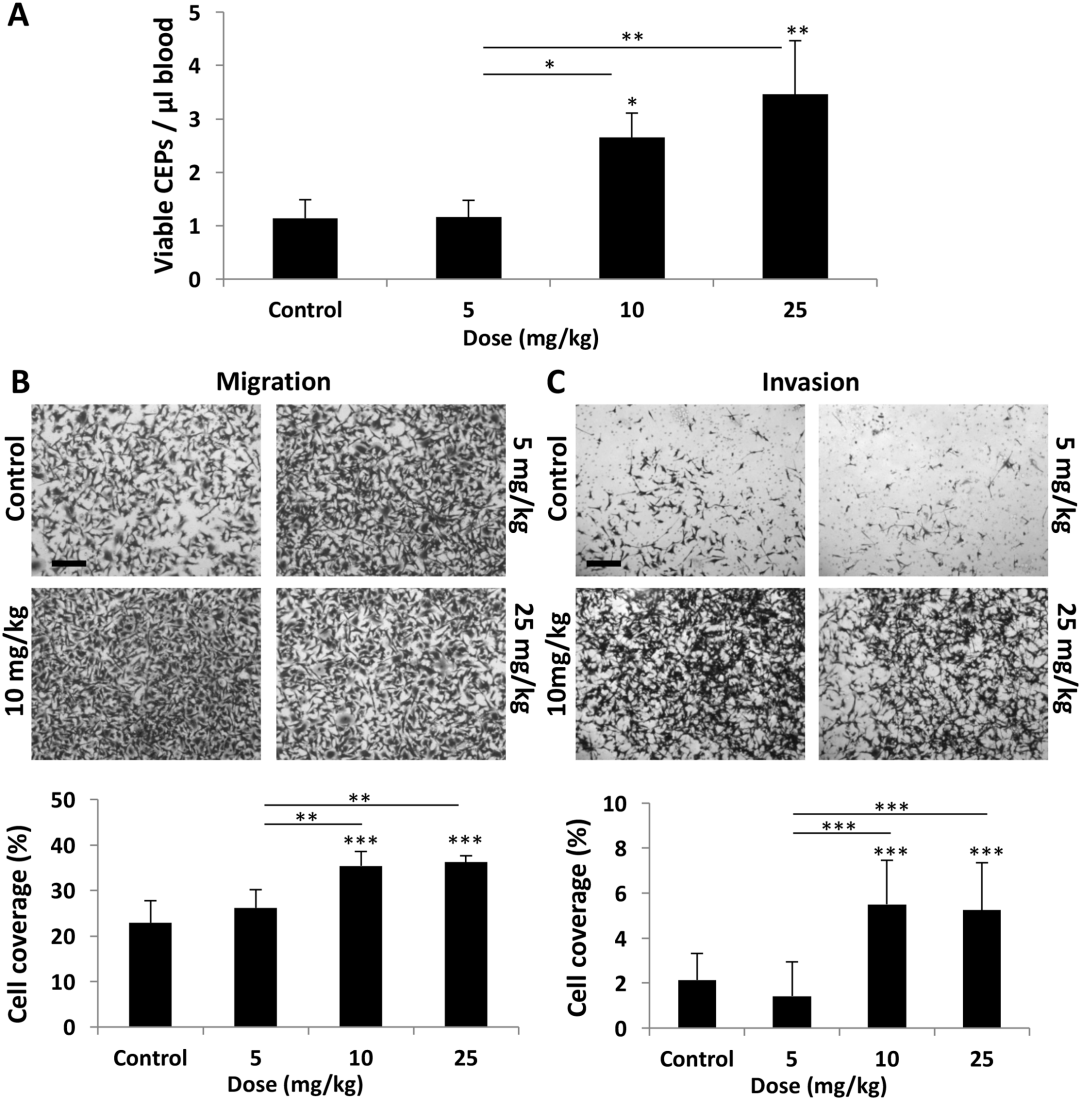
Host response following PTX chemotherapy is dependent on drug dose **(A-C)** Eight-to-ten week old non-tumor bearing BALB/c mice were treated with escalating doses of paclitaxel ranging from 0-25 mg/kg. (A) After 24 hours, blood was drawn by cardiac puncture, and subsequently assessed for the levels of viable CEPs using flow cytometry. (B-C) In parallel, plasma was separated and placed in the bottom compartment of a Boyden chamber to assess MDA-MB-231 cell migration (B) and invasion (C) properties as described in Materials and Methods. Representative images are provided for migration and invasion. Percentage of cell coverage was calculated using Photoshop. Scale bar=200μm. ^*^p<0.05; ^**^p<0.01; ^***^p<0.001 using one way ANOVA followed by Tukey post-hoc test.

To characterize the differential host-mediated responses to chemotherapy, we first evaluated specific elements previously reported at the host, 24 hours after the administration of escalating doses of PTX. We focused on levels of viable CEPs [3] as well as migration and invasion of tumor cells in the presence of plasma obtained from PTX-treated mice [8]. To this end, non-tumor bearing BALB/c mice were treated with PTX at doses of 5, 10, and 25 mg/kg. After 24 hours, blood was drawn by cardiac puncture and the level of viable CEPs was analyzed. Figure 1A demonstrates that the level of viable CEPs was significantly elevated in response to 10-25 mg/kg, but not 5 mg/kg PTX. In addition, the migration and invasion properties of MDA-MB231, human breast carcinoma cells, were enhanced in the presence of plasma from mice treated with 10-25 mg/kg PTX, whereas plasma from mice treated with 5 mg/kg PTX had no effect (Figure 1B–1C).

**Figure 2:**
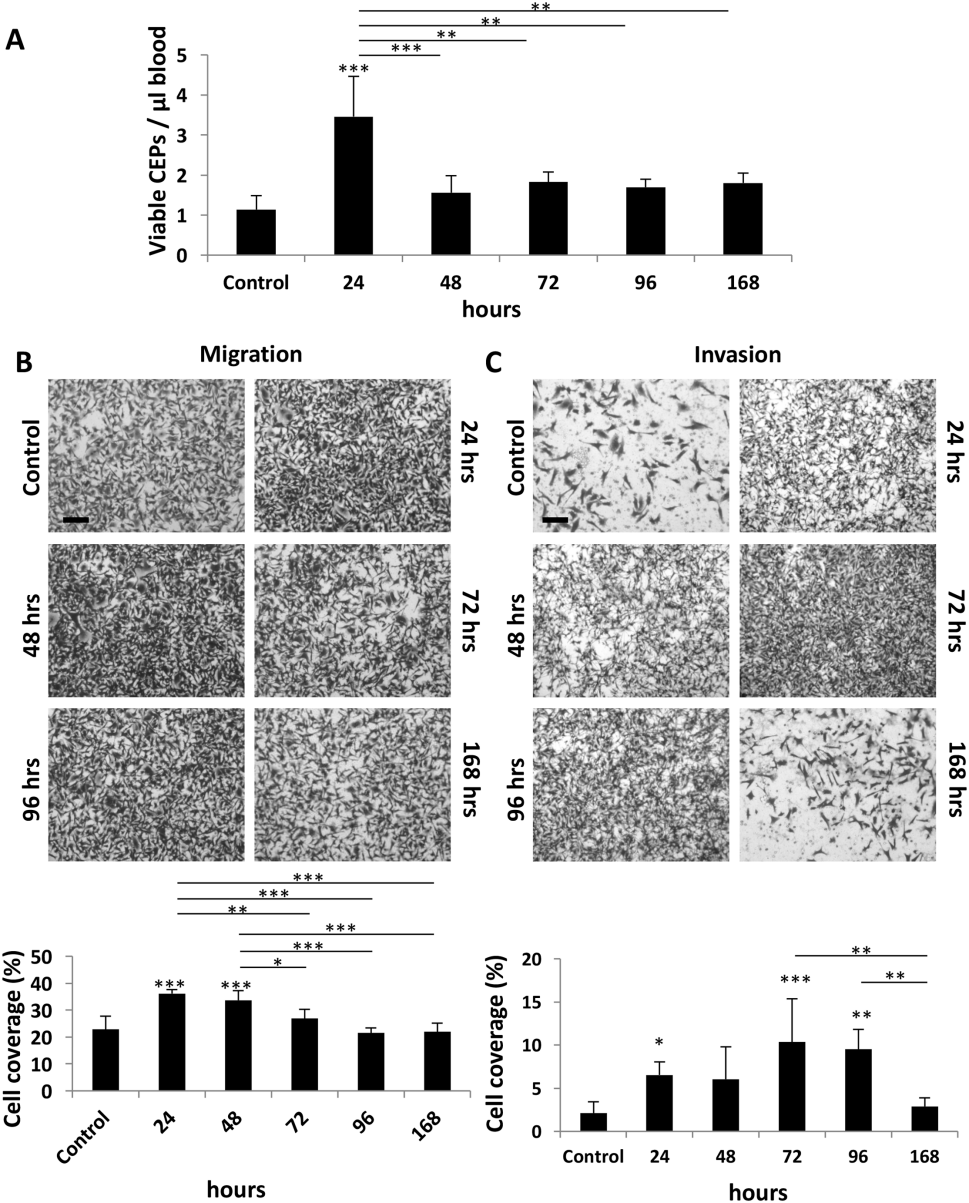
Host response following PTX chemotherapy is time-dependent **(A-C)** Eight-to-ten week old non-tumor bearing BALB/c mice were treated with 25 mg/kg paclitaxel. After 24, 48, 72, 96, and 168 hours blood was drawn by cardiac puncture, and subsequently assessed for the levels of viable CEPs using flow cytometry. (B-C) In parallel, plasma was separated and placed in the bottom compartment of a Boyden chamber to assess MDA-MB-231 cell migration (B) and invasion (C) properties as described in Materials and Methods. Representative images are provided for migration and invasion. Percentage of cell coverage was calculated using Photoshop. Scale bar=200μm. ^*^p<0.05; ^**^p<0.01; ^***^p<0.001 using one way ANOVA followed by Tukey post-hoc test.

We next determined the duration of the host response after administering PTX at a dose of 25 mg/kg. This represents the MTD, as previously described [3]. Specifically, we evaluated to what extent the host effects are maintained following drug administration. Levels of CEPs peaked at the 24 hour time point, returning to baseline within 48 hours (Figure 2A). In addition, the migration properties of MDA-MB231 cells were enhanced in the presence of plasma derived from mice up to 48 hours after PTX treatment (Figure 2B). However, plasma derived from mice up to 96 hours after PTX treatment enhanced the invasive properties of such cells (Figure 2C). The differential duration of specific host effects suggests that they are regulated by different pathways or factors.

### Mathematical modeling rejects an explanation of host effect dynamics on invasion, based on classical pharmacokinetic/pharmacodynamic mechanisms

**Figure 3:**
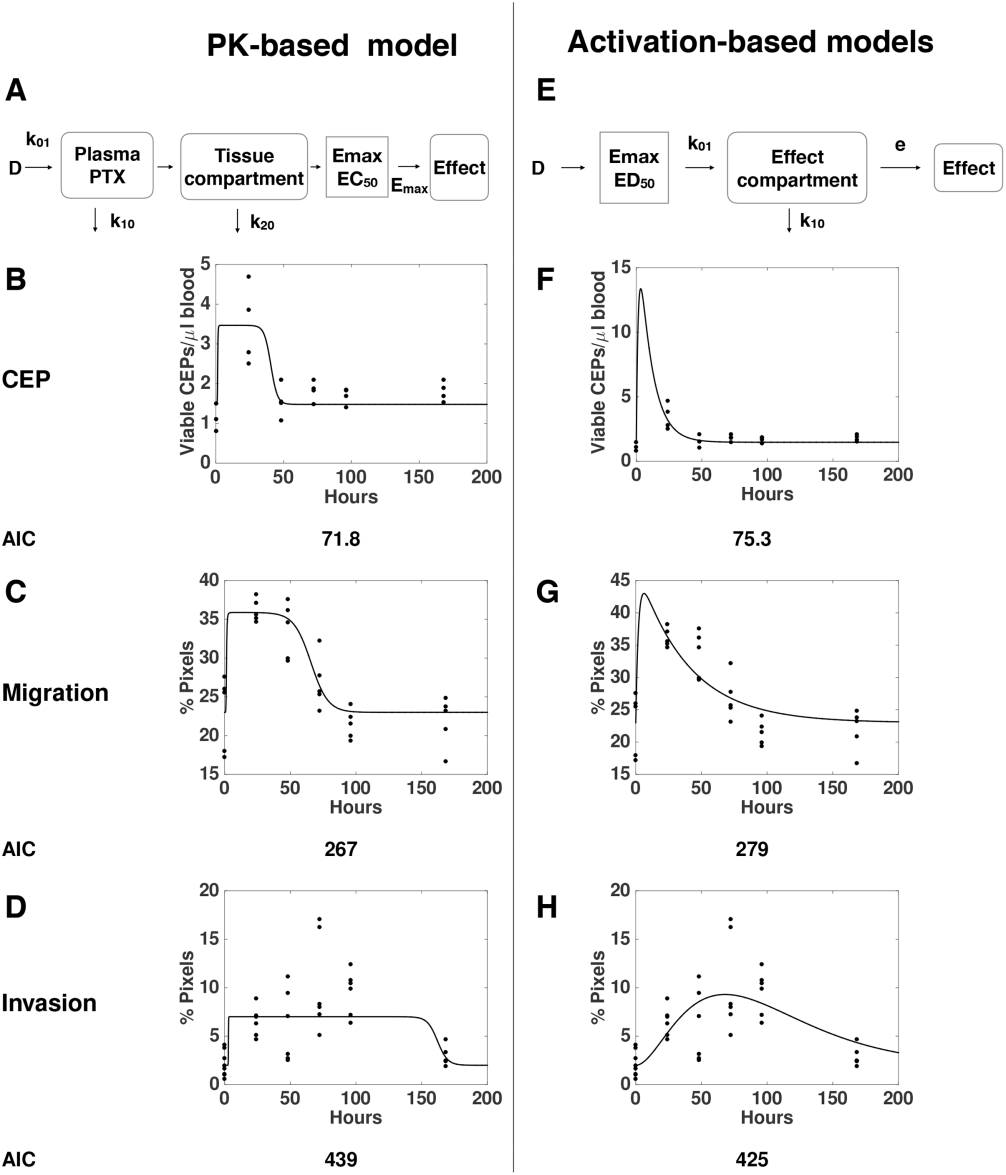
“Pharmacokinetics (PK)-based” versus “Activation-based” models Mathematical models based on distinct mechanisms: PK-based **(A-D)** versus activation-based **(E-H)**. Schemes of the mathematical models for PK-based (A) versus activation-based (E) are presented. The mathematical models were formulated for description of the dynamics of host effects characterized by levels of viable CEPs (B, F) as well as migration (C, G) and invasion (D, H) assays. Confrontation of the models to the data through likelihood maximization supported a standard PK/Emax-based model for the migration and viable CEP effects but rejected it in favor of an activation-based mechanism for the invasion data. Dots = data, lines = models best-fits (see materials and methods).

To quantitatively assess various mechanistic models for the dynamics of the processes leading to these observed host-mediated effects, we designed several mathematical models and tested their descriptive properties against the data. Pharmacological drug effects can be traditionally modeled in a two-step approach (Figure 3A) [23]. First, the dynamics of the drug distribution, metabolism and elimination is described (pharmacokinetics, PK). Then, the link between the concentration of the drug and the effect is modeled (pharmacodynamics, PD). For the latter, a saturable Emax model (or Hill function) is classically employed [23]. When testing this approach against our data – using a published model and parameter values for PTX pharmacokinetics [24], and separate models for each effect - we found that the models with Emax effects that are directly linked to the circulating PTX concentration, were unable to correctly describe our data (Supplementary Figure 1). Addition of effect compartments was able to give a correct description of the CEP and migration effects (Figure 3B–3C). However, critically, this approach was unable to explain the combined dose-dependence and dynamics of the invasion effects (Figure 3D). This counter intuitive finding comes from the opposition between two observations on the invasion data that are mutually exclusive under the classical PK/PD paradigm. On one hand, the function of effect versus dose (Figure 1C) strongly suggests a saturation of the effect above a given threshold (between 5 and 10 mg/kg). This translates into an Emax PD model. However, the function of effect versus time is not consistent with this assumption, because a saturation should be apparent (when the concentration is above the PD threshold), in contradiction with the actual observations (Figures 2 and 3D). Maximization of the data likelihood under two models – the classical PK-based model and an alternative, “activation-based” model – clearly rejected the former in favor of the latter for the invasion data (Akaike Information Criterion, AIC = 439 vs 425). This prompted us to propose another model based on the assumption that the effect is triggered directly by the dose (and not by the PK-described concentration of the drug), and then follows compartmental kinetics (see Materials and Methods and scheme in Figure 3E). While this new model was less likely for the host-mediated effects on CEP levels and migration assays (Figure 3F–3G, AIC = 71.8 versus 75.3 and 267 versus 279 in comparing the PK-based model versus the activation-based one, respectively for CEP levels and migration effects), it offers a more accurate description of the dose- and time-dependence of the effect on invasion (Figure 3H and Supplementary Figure 2). Together, these results support different mechanisms for the migration and CEP effects on one hand, and the invasion effect on the other, and evidenced a counter-intuitive model for the latter.

### A global model for the dynamics of host-mediated effects

**Figure 4:**
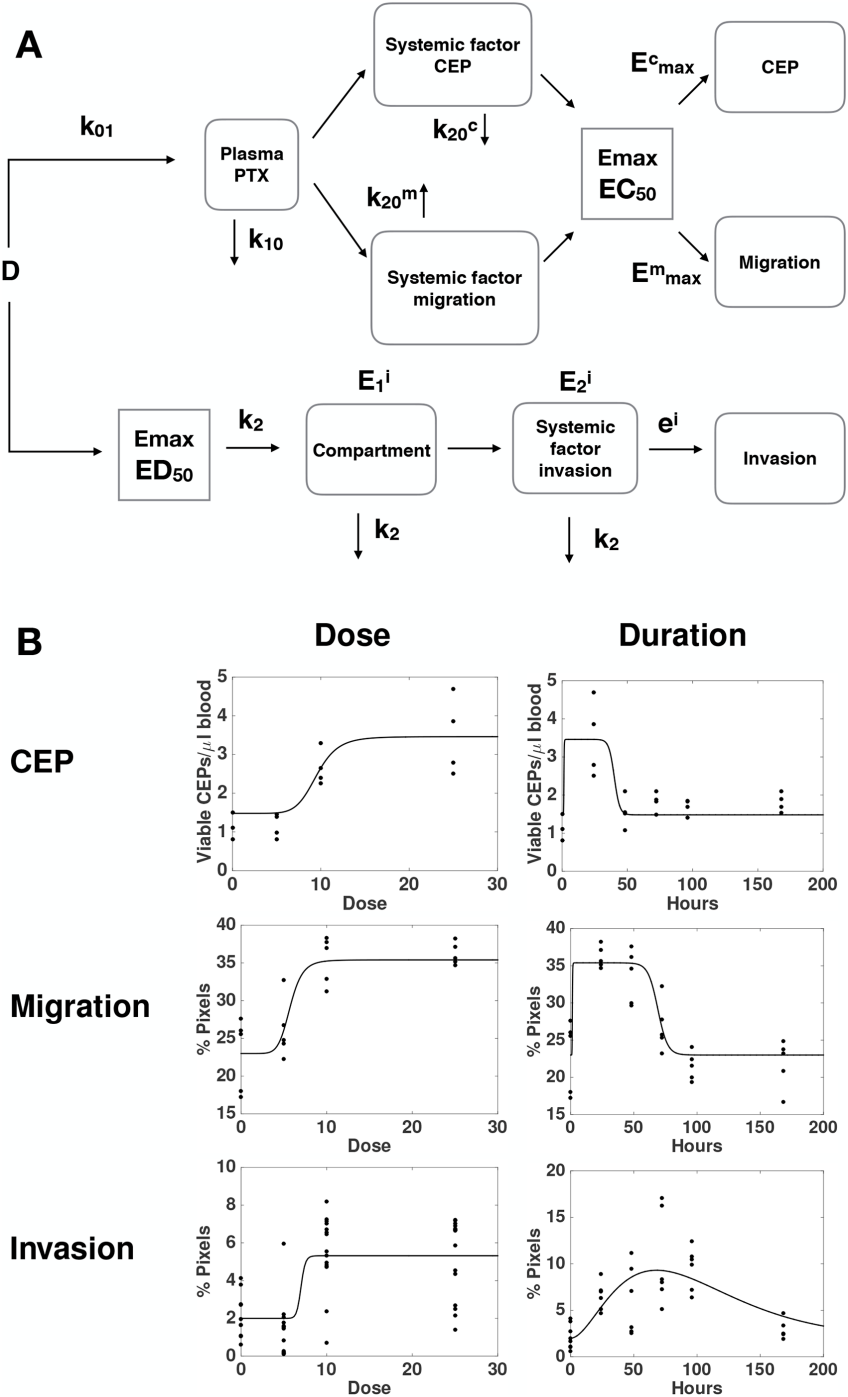
A global mathematical model for the combined dynamics of all host-mediated effects **(A)** Formulation of a semi-mechanistic model was able to **(B)** reproduce the data as function of the dose and post-PTX administration time.

To further investigate the mechanisms of the distinct host-mediated effects in response to PTX, as being provoked by either a single or multiple factors, we investigated global mathematical models that would explain all the data together, i.e., the three effects monitored here. Maximization of the likelihood was thus performed by pooling all the data from the three readouts considered here, instead of independently as performed above. Since we had previously identified different mechanisms between the effect on invasion and the other two variables tested, we focused on different possibilities for structural links between only the effects on migration and CEP levels. From a restrictive model, where the effect on migration and CEP levels would be tied to the same factor (model 1, Supplementary Figure 3), we considered the following extensions with distinct factors for migration and CEP levels and: 1) same elimination rate from the tissue compartment *k*_20_ but distinct *EC*_50_ (model 2, Supplementary Figure 4); 2) same *EC*_50_ in the PD Emax model but distinct *k*_20_ (model 3, Figure 4); and 3) distinct *k*_20_ and *EC*_50_ (model 4, Supplementary Figure 5). Results of goodness-of-fit statistical metrics are reported in Table 1. Likelihood ratio tests, indicating whether a model with more parameters – while always having better goodness-of-fit by definition – is significantly more likely, are shown in Supplementary Table 1. They demonstrate that model 1 was clearly outperformed by models 2, 3 and 4 (p < 0.0001, likelihood ratio tests against models 1-3). Consideration of distinct *EC*_50_ (model 2) for factors responsible for the effects on migration and CEP levels significantly improved the fit (model 2 versus model 1, p < 0.0001). The best improvement was provided by distinction in the elimination rate *k*_20_. Indeed, this addition to model 2 significantly improved the fit further (p < 0.024, model 4 versus model 2). In fact, this addition alone, model 3, despite having less parameters (and thus a larger log-likelihood), was found to be significantly more likely than model 4 (smaller AIC and BIC, Table 1 and non-significant likelihood ratio test for superiority of model 4, Supplementary Table 1). Therefore, we selected model 3 (see scheme in Figure 4A) as the best candidate for the description of our data (Figure 4B). Together, these results further support a model with distinct quantitative dynamics between all the host responses (but equal activation threshold for the factor responsible for effects on CEP levels and migration), suggesting that distinct factors are responsible for these effects.

**Table 1:** Goodness-of-fit criteria for the models of host response dynamics

Model	Log.lik.	AIC	BIC	#
Model 3	-366.22[2]	**750**[1]	**779**[1]	8
Model 4	**-366.18**[1]	752[2]	784[3]	9
Model 2	-368.73[3]	755[3]	784[2]	8
Model 1	-377.21[4]	770[4]	796[4]	7

Log. Lik. = log-likelihood. AIC = Akaike Information Criterion. BIC = Bayesian Information Criterion.

### Validation of the host response mathematical prediction and its *in vivo* outcome

**Figure 5:**
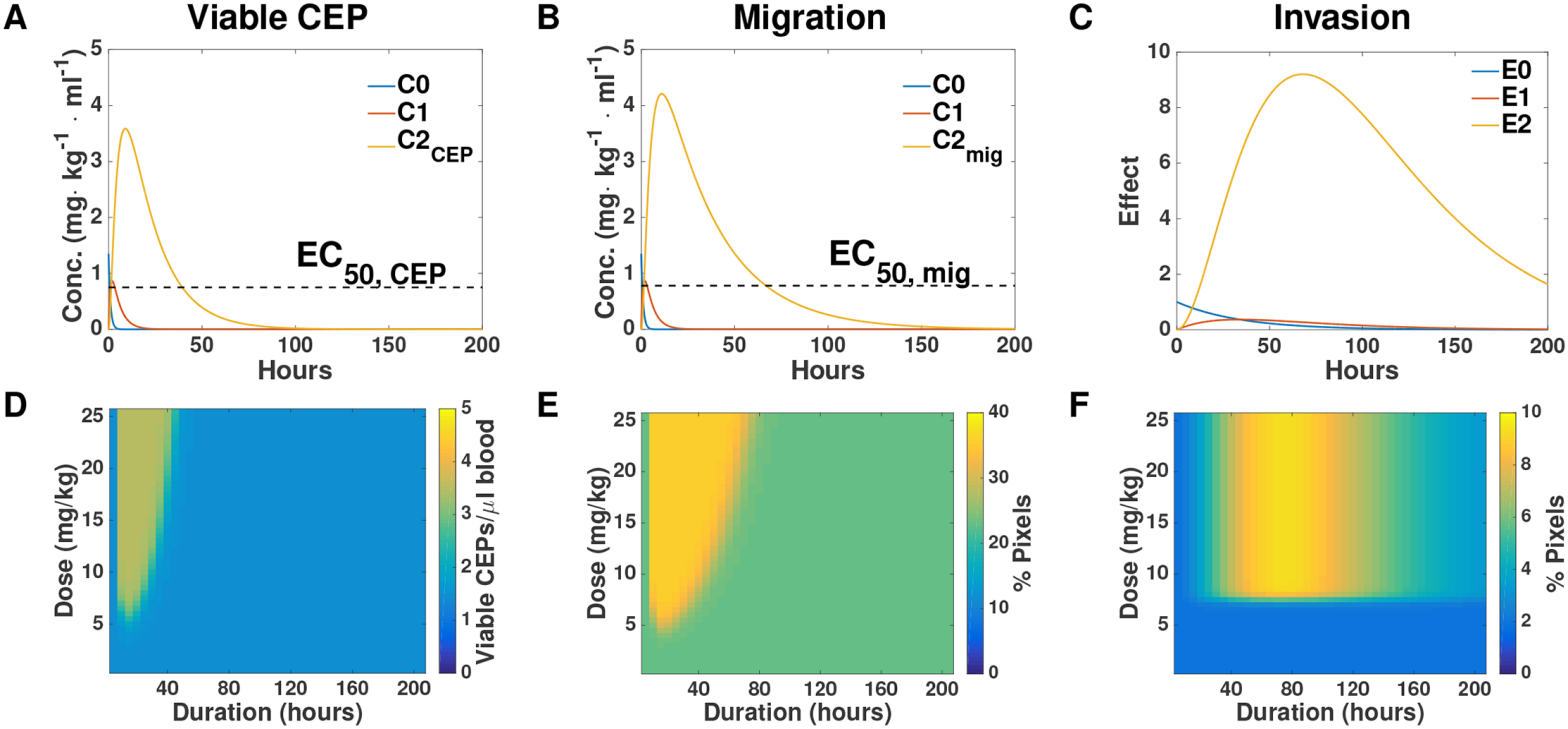
Simulation-based inference and predictions **(A-C)** Simulations of the entire system based on the parameters of viable CEP (A), migration (B), and invasion (C) calibrated from the data. **(D-F)** Predictions of the intensity of host response effects in dose-duration space, based on the parameters of viable CEP (D), migration (E), and invasion (F). Conc. = concentration.

Our mathematical model allowed us to infer the dynamics of the hypothesized hidden quantities responsible for the measured effects. Simulations of these, based on the parameters calibrated from the data reported in the Supplementary Table 2, are shown in Figure 5A-5C. Going further, the model allowed us to make predictions about host effects over all doses and durations, thus extending the experimental findings. Figure 5D-5F reports the predicted effects on CEP level, migration, and invasion for combinations of doses spanning 0-25 mg/kg and 0 – 200 hours following PTX administration. Structural differences between the PK-based models for the migration and CEP effects on one hand, and the activation-based models on the other, can be observed. For the former, a duration-dependent dose threshold is apparent (Figure 5A–5B). This means that at larger times, similar effects can be observed for a larger dose. Conversely, for the latter, a duration-independent dose threshold is apparent (Figure 5C). This means that for each duration, the effect – small or large – happens after the same threshold of 7 mg/kg. Similarly, in PK-based models the duration of the effect is dose-dependent whereas in the activation-based model it is not.

**Figure 6:**
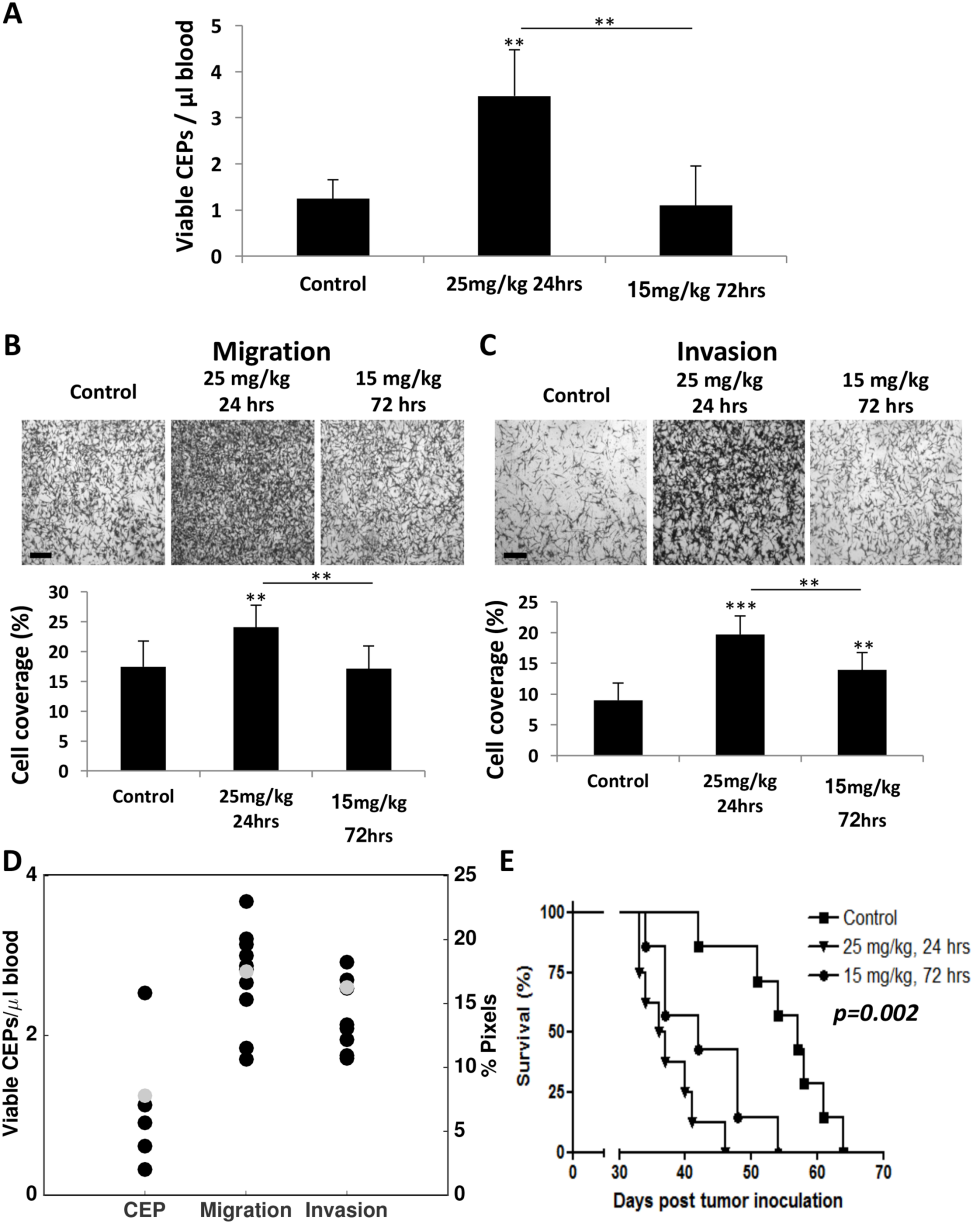
Differential host response following paclitaxel chemotherapy results in diverse survival outcome **(A-C)** Eight-to-ten week old non-tumor bearing BALB/c mice were treated with 25 mg/kg or 15 mg/kg paclitaxel. After 24 and 72 hours, respectively, blood was drawn by cardiac puncture, and subsequently assessed for the levels of viable CEPs using flow cytometry. **(B-C)** In parallel, plasma was separated and placed in the bottom compartment of a Boyden chamber to assess MDA-MB-231 cell migration (B) and invasion (C) properties as described in Materials and Methods. Representative images are provided for migration and invasion. The percentage of cell coverage was calculated using Photoshop. Scale bar=200μm. **(D)** Comparison of experimental data and model predictions for host-mediated effects 72 hours after injection of 15 mg/kg PTX. Black dots = data. Gray dots = model predictions. **(E)** Ten week old NOD-SCID female mice were treated with 25 mg/kg or 15 mg/kg paclitaxel. After 24 and 72 hours, respectively, MDA-MB-231 cells (2×10^6^ cells/mouse) were intravenously injected through the tail vein. A Kaplan-Meier survival curve is shown. ^**^p<0.01; ^***^p<0.001 using one way ANOVA followed by Tukey post-hoc test.

To assess whether the model predicting host response effects is valid, we next evaluated the host response at different time points after administrating two concentrations of PTX and compared the experimental data with the mathematical model's prediction. To this end, BALB/c mice were treated with either 25 mg/kg and blood was drawn after 24 hours or with 15 mg/kg and blood was drawn after 72 hours. The levels of viable CEPs rose only at the dose of 25 mg/kg after 24 hours, whereas no changes were observed at the dose of 15 mg/kg at the 72 hour time point (Figure 6A). Furthermore, at the 25 mg/kg dose at 24 hour time point, both migration and invasion were significantly higher compared to baseline or to the dose of 15 mg/kg dose at 72 hours. Notably, the dose of 15 mg/kg at 72 hours was still significantly higher than baseline control only in the invasion assay (Figure 6B–6C). Model predictions of the effects at the new dose of 15 mg/kg and after 72 hours (with baseline values *ε*^i^, *ε*^m^ and *ε*^c^ adjusted to the mean of the control values for this new experiment) were found in excellent agreement with the observations (Figure 6D). This result strengthens the validity of our model as a useful tool to predict host effects for values of dose and duration that were not used for calibration of the parameters.

Next, our validation experiment results prompted us to further assess whether activation of the host response affects survival. Since increased CEP levels in response to therapy are associated with angiogenesis [9] and enhanced migration and invasion properties of tumor cells are associated with tumor cell aggressiveness [10, 25], such effects are expected to increase the mortality rate in experimental lung metastasis assays, as previously reported [8, 26]. In order to separate the therapeutic effect of the treatment from the host-mediated effects, we treated NOD-SCID mice with PTX chemotherapy and subsequently injected MDA-MB-231 cells (2×10^6^ cells/mouse) through the tail vein to generate an experimental lung metastasis assay. The NOD-SCID mice were either treated with 25 mg/kg PTX and injected with tumor cells 24 hours later or with 15 mg/kg PTX and injected with tumor cells 72 hours later. Survival was monitored over time. The results in Figure 6E demonstrate that mice from the first group succumbed to metastasis earlier than mice from the second group. The survival plots of these two treated groups were significantly different (p=0.048). Importantly, mice from both PTX treated groups exhibited an increased mortality rate compared to untreated control mice (p=0.002), indicating that the host-mediated effects in response to PTX are pro-tumorigenic as previously shown [8]. Taken together, our improved mathematical model was able to predict the parameters of host-mediated effects that were measured. Such host effects were correlated with the survival outcome of mice treated with different PTX doses and at two time points following drug administration.

## DISCUSSION

Chemotherapy is one of the main treatment modalities for cancer. While treatment benefit is usually observed, some patients relapse due to the development of therapy resistance. The resistance of tumor cells to therapy is the most studied mechanism underlying relapse [27]; however, recently, the contribution of the host to therapy resistance, especially after chemotherapy, has gained more attention [28]. It has been shown that such host effects in response to therapy minimize or even negate the anti-tumor activity of chemotherapy, thereby providing another route for resistance mechanisms [1].

Macrophages, fibroblasts and mesenchymal stem cells have been shown to contribute to tumor resistance by secreting factors, some of which are associated with fatty acids and extracellular matrix (ECM) degrading enzymes, or by promoting an enrichment of cancer stem cells [5, 29, 30]. In addition to these mechanisms, we and others have demonstrated that various treatment modalities for cancer support tumor cell aggressiveness. For example, bone marrow derived cells home to the treated tumor site, and secrete MMP9 which contributes to the dissemination of tumor cells from the primary tumor and induces epithelial-to-mesenchymal transmission (EMT) in tumor cells, thereby accelerating metastasis. Furthermore, bone marrow derived CEPs are acutely mobilized in response to high dose chemotherapy, and subsequently home to the treated tumor site, leading to rapid angiogenesis and subsequent tumor re-growth [3, 9, 31]. Thus, the host response to chemotherapy activates a variety of tumor-supporting processes, leading to increased aggressiveness and re-growth.

Several studies have reported a distinct host response following MTD chemotherapy, an effect that is absent when the same chemotherapy drug is administered in LDM regimens. Specifically, the increase in levels of CEPs in peripheral blood or the number of myeloid derived suppressor cells colonizing tumors following MTD regimens is absent when LDM regimens are employed [32, 33]. Additionally, tumor cells cultured in the presence of plasma from mice treated with high-dose capecitabine exhibited distinct tumor sphere characteristics, whereas plasma from the low-dosage-treated mice had no such effect [15]. These collective studies indicate that MTD but not LDM chemotherapy regimens generate host pro-tumorigenic and pro-metastatic effects. In fact, with respect to LDM chemotherapy, not only are there no detectable host pro-tumorigenic and pro-metastatic effects, such therapy may actively contribute to anti-tumor activity by other means. For example, it has been suggested that LDM therapy induces the immune system to act against cancer cells. LDM therapy also depletes T regulatory cells, and therefore the combination of LDM chemotherapy with immune checkpoint inhibitors has been suggested as an effective treatment combination for cancer [34–36]. In addition, it has been demonstrated that long exposure of tumor cells to chemotherapy drugs at low doses directly kills cancer cells, among them cancer stem cells [37, 38]. Thus, LDM therapy acts against the tumor via various host and tumor dependent mechanisms. It should be noted, however, that while our study evaluated the host response effects following a range of doses of PTX therapy, we have not evaluated the effect of continuous administration of a drug as administrated in LDM regimen in comparison to a single MTD administration. Future studies should assess our mathematical model in this context.

In this study, we have formulated a mechanistic mathematical model to solely predict pro-tumorigenic and pro-metastatic effects generated by the host in response to therapy, while eliminating from the formula the direct anti-tumor activity of the drug. As a readout for the host response to therapy, we evaluated three previously characterized variables [3, 8], namely CEP levels in peripheral blood and the effect of plasma derived from chemotherapy-treated mice on migratory and invasive properties of tumor cells *in vitro*. The variables were tested at different doses of chemotherapy and at specified time-points after drug administration. Our results show that doses below a specific threshold do not generate a host response and therefore are less likely to contribute to tumor cell aggressiveness. Above this dose threshold, measurements of the host response are saturated to maximal values. Classically, such a saturation can be modeled by means of *E_max_* models [23], which when coupled to pharmacokinetics models for the concentration time course, were indeed found appropriate for the CEP and migration variables. Counter-intuitively however, when modeling concurrently the time-course of the effect and the dose dependence, for the invasion assays, a model based on this approach was found unable to adequately reproduce the data. Instead, an “activation-based” model was proposed where the dose (instead of the concentration) directly triggers the effect. Predictions of these two different models – activation-based versus pharmacokinetics-based – differ as follows: in the first case, the duration of the effect mostly depends on the dose being below or above the threshold, while in the second case, the duration increases with increasing dosage. Investigating further a global model including all the host effects, we found that the migration and CEP variables, while relying on the same structural model, had different coefficients (and thus different durations). The generalizability of this mathematical model to additional doses and durations was validated by comparing predictions of the model with experimental results at a different dose and time point. Importantly, we next validated our mathematical model by evaluating tumor cell aggressiveness *in vivo*. To do so, we used an experimental lung metastasis model in mice ‘pre-conditioned’ with different doses of PTX chemotherapy and injected with tumor cells at different time points following drug administration. Our previous study indicated that such an approach solely evaluates the effect of the host in response to chemotherapy [8], as opposed to the tumor-mediated response of spontaneously growing tumors treated with chemotherapy.

Pharmacokinetic-pharmacodynamic approaches in oncology have so far mostly focused on modeling hematological toxicities and few of them have been dedicated to efficacy [39, 40]. Methods employed for modeling the efficacy of cytotoxic drugs have linked the effect to the drug concentration by means of well-established differential equations for description of tumor growth [18]. Apart from the classical log-kill Skipper-Schabel-Wilcox [41] and Norton-Simon [42] models, more recent works include: transit compartments modeling damaged cancer cells [43, 44], an interface effect compartment [45] or models specific to metronomic chemotherapy based on the assumption of an anti-angiogenic action of the cytotoxic drug [20, 21, 46]. Counterintuitively however, when concurrently modeling the time-course of the effect and the dose dependence for the invasion assays, a model based on this approach was unable to adequately reproduce the data. Importantly, this inadequacy with the data was structural and not merely quantitative. No PK-based model would be able to describe this data, as any such model would exhibit a plateau phase, which is absent in our data. Therefore, despite the fact that our PK-based model uses previously published parameter values [24] rather than parameters calibrated from actual concentration data, the conclusion that a PK-based model does not fit the invasion data remains valid. Instead, an ‘activation-based’ model is proposed where the dose directly triggers the effect.

While our study only evaluated the host response to PTX chemotherapy, it would be of interest to evaluate the host response to other chemotherapy drugs or other treatment modalities for cancer. For example, a recent study demonstrated that breast cancer patients treated with neoadjuvant chemotherapy of paclitaxel after doxorubicin plus cyclophosphamide exhibit an increased number and density of tumor microenvironment of metastasis (TMEM), a known prognostic marker of metastasis, despite the decrease in tumor size [47]. In another study, Brown and colleagues recently demonstrated that high-dose radiation (6 Gy) leads to colonization of myeloid cells in the brain supporting angiogenesis and tumor re-growth [48, 49]. In addition, we previously reported that radiation at doses above 6 Gy resulted in the mobilization of CEPs [50]. Furthermore, a study by Bertolini and colleagues reported that circulating angiogenic factors and bone marrow derived cells are acutely mobilized in patients who underwent open surgery when compared to minimally invasive surgery, namely, laparoscopy [51]. Thus, these aforementioned studies demonstrate that the efficacy of various treatment modalities for cancer is dependent, in part, on host-generated pro-tumorigenic effects. Specifically, we suggest that the anti-tumor effects of the drug are, to some extent, counteracted by host-mediated effects that promote tumor cell aggressiveness, ultimately contributing to tumor relapse and metastasis. Therefore, measuring such effects, or developing mathematical models for these effects, may help predicting therapeutic efficacy.

Clinically, several previous studies have investigated whether chemotherapy dose adjustment can affect therapeutic outcome in cancer patients. The method of adjustment was based on personalized tolerability of an individual patient to MTD chemotherapy as assessed by the degree of toxicity (i.e., side effects) or pharmacokinetic measurements. For example, in a retrospective study of capecitabine therapy for metastatic breast cancer patients, dose adjustment based on adverse events did not compromise therapeutic efficacy [52]. In another study of methotrexate for the treatment of childhood acute lymphoblastic leukemia, a comparison between fixed-dose chemotherapy and individualized-dose based on drug concentration in plasma, resulted in better outcome in those patients in the individualized therapy arm [53]. In addition, it has also been demonstrated in several clinical pharmacokinetic studies that even though LDM therapy is administered at low-doses, active drug concentrations can be achieved by such therapy owing to the continuous administration of the drug [54]. Thus, it is critical to assess the optimal dosage of drugs in a personalized manner in cancer patients [55]. We propose that the host response to therapy could be used as an additional parameter in order to define the optimal dose to enhance therapeutic outcome.

The differential effects on CEP mobilization, and tumor cell migration, and invasion at different time points following drug administration suggest that expression of a diverse range of factors contribute to the host response to therapy. Several previous studies reported that G-CSF and SDF-1, for example, are factors involved in the acute mobilization of CEPs in both patients and mice in response to MTD chemotherapy [3, 56]. In addition, intrinsic and extrinsic tumor cell expressing factors affect migration and invasion properties. MMPs and other ECM proteolytic enzymes secreted by tumor and host cells contribute to ECM degradation leading to enhanced invasion properties of tumor cells [57]. Furthermore, increased levels of epidermal growth factor (EGF), insulin growth factor-1 (IGF1) and lysophophatidic acid (LPA) have been shown to promote tumor cell pro-migratory effects [25]. Thus, such factors present in the plasma represent candidates that are most likely affected by the therapy. The question is whether the circulating levels of these different factors are altered in response to different doses of therapy or over time after drug administration. It would be of interest to evaluate the dynamics of some of these factors and assess whether they correlate with the dynamics of specific host responses assessed in this study.

In summary, our study describes the process of identifying a proper mathematical equation to adequately formulate various host-mediated effects in response to chemotherapy drugs. This model, which is based on three specific host-mediated effects, can be applied to other chemotherapy drugs. Using this model we were able to adequately predict host-mediated effects in response to different doses of drugs administered in mice. We propose that this mathematical tool may aid in the design of appropriate chemotherapy regimens for cancer patients in order to improve therapeutic outcome.

## MATERIALS AND METHODS

### Equations and assumptions

#### Independent models

These models were designed to describe each of the host-mediated effects separately and consisted of two classes: pharmacokinetics-based or activation-based models.

*Pharmacokinetics-based models* relied on the following assumptions (see scheme in Figure 3A):

(PH1) The PTX concentration follows a one- or two-compartmental distribution kinetics with the administered dose as input [23].

(PH2) The effect is tied to the *concentration* of PTX (in either the plasma or some other tissue compartment) through a Hill function (also called Emax) dependence [23].

A one-compartment pharmacokinetics model was quickly disregarded (see results) and thus we only report on the model with two compartments (plus a usual absorption compartment).

The corresponding equations write:

$$\left\{ \begin{array}{c} \begin{array}{ll} \frac{dC_{0}}{dt}=-k_{01}C_{0} & C_{0}\left(0\right)=\frac{D}{V_{d}}\\ \frac{dC_{1}}{dt}=k_{01}C_{0}-k_{10}C_{1} & C_{1}\left(0\right)=0\\ \frac{dC_{2}}{dt}=C_{1}-k_{20}C_{2} & C_{2}\left(0\right)=0 \end{array}\\ E\left(t,D\right)=\epsilon +(E_{max}-\epsilon )\frac{C_{2}{\left(t\right)}^{\gamma }}{EC_{50}^{\gamma }+C_{2}{\left(t\right)}^{\gamma }} \end{array}\right.$$

The variables are:

The time *t* (= duration following injection of PTX); the dose *D*, the PTX concentrations in the absorption (*C*_0_), central (*C*_1_) and tissue (*C*_2_) compartments; and the effect *E*.

The parameters of these models (specific to each experimentally measured effect) are:

The volume of distribution of the central compartment *V*_d_; the compartment transition and elimination rates *k*_01_, *k*_10_ and *k*_20_; and the Emax model effect parameters, baseline effect *ε*, maximal effect *E_max_* concentration needed to have half of the maximal effect *EC*_50_ and Hill coefficient *γ*.

In contrast, *activation-based models* are based on the reverse mechanism (Figure 3E):

(AH1) The effect is triggered directly by the *dose* (in contrast to the concentration), *via* an Emax dependence.

(AH2) Once activated, the effect follows a two-compartmental kinetics.

The corresponding equations write:

$$\left\{ \begin{array}{c} E_{0}^{0}\left(D\right)=\epsilon +(E_{max}-\epsilon )\frac{D^{\gamma }}{ED_{50}^{\gamma }+D^{\gamma }}\\ \begin{array}{ll} \frac{dE_{0}}{dt}=-k_{01}E_{0} & E_{0}\left(0\right)=E_{0}^{0}(D)\\ \frac{dE_{1}}{dt}=k_{01}E_{0}-k_{10}E_{1} & E_{1}\left(0\right)=0\\ \frac{dE_{2}}{dt}=E_{2}-k_{20}E_{2} & E_{2}\left(0\right)=0 \end{array}\\ E=eE_{2} \end{array}\right.$$

The dynamic variables are now *E*_0_, *E*_1_ and *E* for description of the effect (or the dynamics of systemic factors following activation of the host response). The parameters have similar interpretation as in the above model, except *ED*_50_, which is now a dose-dependent threshold and an additional efficacy parameter *e*.

#### Global model

The global model (Figure 4A) was built on PK-based models for the migration and CEP effects – relying both on the paclitaxel blood concentration – and activation-based dynamics for the invasion effect. The equations write:

$$\{\begin{array}{ll} \frac{dC_{0}}{dt}=-k_{01}C_{0} & C_{0}\left(0\right)=\frac{D}{V_{d}}\\ \frac{dC_{1}}{dt}=k_{01}C_{0}-k_{10}C_{1} & C_{1}\left(0\right)=0\\ \frac{dC_{2}^{m}}{dt}=C_{1}-k_{20}^{m}C_{2}^{m} & C_{2}^{m}\left(0\right)=0\\ \frac{dC_{2}^{c}}{dt}=C_{1}-k_{20}^{c}C_{2}^{c} & C_{2}^{c}\left(0\right)=0\\ E^{m}\left(t,D\right)=\epsilon^{m}+(E_{max}^{m}-\epsilon^{m})\frac{C_{2}^{m}{\left(t\right)}^{\gamma }}{{\left(EC_{50}^{m}\right)}^{\gamma }+C_{2}^{m}{\left(t\right)}^{\gamma }} & \\ E^{c}\left(t,D\right)=\epsilon^{c}+(E_{max}^{c}-\epsilon^{c})\frac{C_{2}^{c}{\left(t\right)}^{\gamma }}{{\left(EC_{50}^{c}\right)}^{\gamma }+C_{2}^{c}{\left(t\right)}^{\gamma }} & \\ E_{0}^{0}\left(D\right)=\frac{D^{\gamma }}{ED_{50}^{\gamma }+D^{\gamma }} & \\ \frac{dE_{0}^{i}}{dt}=-k_{2}E_{0}^{i} & E_{0}^{i}\left(0\right)=E_{0}^{0}(D)\\ \frac{dE_{1}^{i}}{dt}=k_{2}E_{0}^{i}-k_{2}E_{1}^{i} & E_{1}^{i}\left(0\right)=0\\ \frac{dE_{2}^{i}}{dt}=E_{1}^{i}-k_{2}E_{2}^{i} & E_{2}^{i}\left(0\right)=0\\ E^{i}=\epsilon^{i}+e^{i}E_{2}^{i} & \end{array}$$

We found that having the same parameter for the absorption, elimination in the two compartments and transfer kinetics for the invasion effect, was able to adequately describe the data. Consideration of different parameters resulted in poor identifiability and thus we considered the same transfer rate *k*_2_.

### Cell lines and tissue culture procedures

MDA-MB-231 human breast carcinoma cell line was purchased from the American Type Culture Collection (ATCC, USA), and was used within 6 months of resuscitation. The cell line was authenticated by the genomic center at the Biomedical Core Facility, Rappaport faculty of medicine (Technion). The cells were also tested to be negative for mycoplasma. Cells were cultured in Dulbecco's modified eagle medium (DMEM) supplemented with 10% fetal bovine serum (FBS), 1% L-glutamine, 1% sodium-pyruvate and 1% Penicillin-Streptomycin (Biological Industries, Israel), and cultured at 37°C in 5% CO_2_.

### Animal tumor model and drugs

The use of animals and experimental protocols were approved by the Animal Care and Use Committee at the Technion (ethic number IL0890716). Eight-to-ten week old non-tumor bearing BALB/c mice (Envigo, Israel) were administered intraperitoneally with PTX chemotherapy (TEVA pharmaceutical industries, Israel), at the doses indicated in the text, ranging from 0-25 mg/kg. Blood was drawn after 24-168 hours for the evaluation of viable CEP levels by flow cytometry as described below. In addition, plasma was separated, and subsequently used in migration and invasion assays as described below. In a separate experiment, 10 week old NOD-SCID female mice (breed in house) were used for experimental lung metastasis assay. The mice were injected with PTX at a dose of 25 mg/kg or 15mg/kg and MDA-MB-231 cells (2×10^6^ cells/mouse) were then injected through the tail vain at 24 hours and 72 hours after initial drug administration, respectively. Survival was monitored daily and a Kaplan-Meier survival curve was plotted.

### Flow cytometry

Viable CEP levels in peripheral blood were evaluated by flow cytometry as previously described with some modifications [58]. Briefly, blood was collected from mice by cardiac puncture. Cells were stained with a panel of monoclonal antibodies including anti-CD45 (to exclude hematopoietic cells), anti-VEGF2 and anti-CD31 (endothelial cell markers), and anti-CD117 (a progenitor marker). 7-amino actinomycin-D (7AAD) was used to distinguish between viable and dead cells as previously described [59]. Viable CEPs were defined as CD45^-/dim^/CD117^+^/Flk-1^+^/CD31^+^. After red blood cell lysis, cell suspensions were evaluated by BD LSR-Fortessa (BD Biosciences, San Jose, CA, USA). At least 500,000 events were collected in the overall gates, and at least 150 events were collected in the viable CEP gate. Flow cytometry analysis was performed using FlowJo V10 software (Ashland, OR, USA).

### Migration and invasion assays

The migration and invasion properties of MDA-MB-231 cells were assayed in either fibronectin- or Matrigel-coated Boyden chambers, respectively, using a previously described protocol [8]. Briefly, serum-starved cells (2×10^5^ cells in 0.2 ml medium) were added to the filter that was coated with either fibronectin (10μg/ml) or Matrigel (50μl). The lower chamber was filled with serum-free DMEM that contained 5% plasma from treated mice. After 4 hours (for migration) or overnight (for invasion) incubation, the cells that migrated to the bottom filter, were fixed and stained with Crystal violet. Images were captured using a LEICA DMI 6000B fluorescence inverted microscope per x100 objective-field (Leica Microsystems, Germany). At least 10 fields per group were evaluated. The percentage of positive pixels (representing cells) covering the bottom membrane compartment over the total pixels in the field was calculated using Photoshop SC2 V9.0 (San Jose, CA, USA). Experiments were carried out in triplicate, and were independently performed at least twice.

### Statistical model and fits of the data

Considering a model *M* (*t*, *D*; *θ*) depending on the time *t*, dose *D* and a vector of parameters *θ* on one hand, and on the other hand, data *Y*^i^(*t_j_*, *D_k_*) with *i* the index of the replicate and *j* and *k* the indices of time and dose, respectively, we assumed:

$$\begin{array}{ll} Y^{i}(t_{j},\;D_{k})= & M(t_{j},\;D_{k};\;\theta )+\eta_{i,j,k},\\ & \eta_{i,j,k}∼N(0,\;\sigma^{2}) \end{array}$$

with *η_i,j,k_* accounting for the measurement error and assumed to be normally distributed. Writing the likelihood of all the observations together (i.e. pooling the dose-dependent effect and duration-dependent effect) gives:

$$\begin{array}{l} p\left({\left\{Y^{i}(t_{j},\;D_{k})\right\}}_{i,j,k};\;\theta ,\;\sigma \right)=\\ \frac{1}{{\left(\sigma \sqrt{2\pi }\right)}^{\sum_{k=1}^{K}I_{k}}}\;\frac{1}{{\left(\sigma \sqrt{2\pi }\right)}^{\sum_{j=1}^{J}I_{j}}}\exp\\ \left(\begin{array}{l} -\overset{K}{\underset{k=1}{\sum }}\overset{I_{k}}{\underset{i=1}{\sum }}\frac{{\left(Y^{i}\left(24,D_{k}\right)-M(24,D_{k};\;\theta \right)}^{2}}{2\sigma^{2}}-\\ \overset{K}{\underset{k=1}{\sum }}\overset{I_{k}}{\underset{i=1}{\sum }}\frac{{\left(Y^{i}\left(t_{j},\;25\right)-M(t_{j},25;\;\theta \right)}^{2}}{2\sigma^{2}} \end{array}\right) \end{array}$$

where *I_k_* and *I_j_* are the number of replicates for dose *D_k_* (at fixed time *t* = 24 hours) and time *t_j_* (at fixed dose *D* = 25 mg/kg), respectively. Taking the log of this expression makes the maximization of the likelihood equivalent to maximization of the log-likelihood defined by:

$$\begin{array}{l} l\left(\theta ,\sigma \right)=-\overset{K}{\underset{k=1}{\sum }}\overset{I_{k}}{\underset{i=1}{\sum }}\frac{{\left(Y^{i}\left(24,D_{k}\right)-M(24,\;D_{k};\;\theta \right)}^{2}}{2\sigma^{2}}-\\ \overset{K}{\underset{k=1}{\sum }}\overset{I_{k}}{\underset{i=1}{\sum }}\frac{{\left(Y^{i}\left(t_{j},25\right)-M(t_{j},\;25;\;\theta \right)}^{2}}{2\sigma^{2}}-N_{tot}\log\left(\sigma \sqrt{2\pi }\right) \end{array}$$

The apposite of this function was minimized using the function fminsearch of the Matlab software (Mathworks Inc., version R2015a with optimization toolbox). Statistical analysis of the goodness-of-fit of the mathematical models was performed using previously reported, internally developed, software [20]. Model selection was based on the Akaike and Bayesian Information Criteria (respectively AIC, and BIC), defined by the following formulae [60]:

$$\begin{array}{l} AIC=-2l\left(\hat{\theta ,}\;\hat{\sigma }\right)+2P,\;\\ BIC=-2l\left(\hat{\theta ,}\;\hat{\sigma }\right)+P\log\left(N_{tot}\right) \end{array}$$

where $\hat{\theta }$ and $\hat{\sigma }$ are the parameters estimates from likelihood maximization and *P* is the number of parameters. These criteria allow balancing the pure goodness-of-fit of a model with the number of parameters, thus helping to avoid overfitting. Another measure of significant superiority of a model with more parameters (valid for embedded models) is given by the result of a likelihood ratio test which rejects a null model *M*_1_ in favor of an alternative model *M*_2_ (with *M*_1_ a submodel of *M*_2_) if its associated p-value is lower than a significance threshold, taken here to 0.05. This p-value is computed from the following statistic:

$$D=2\left(l\left(M_{1}\right)-l\left(M_{2}\right)\right)$$

which follows a chi-square distribution with P2−P1 degrees of freedom under the null hypothesis [61].

### Statistical analysis

Data are expressed as mean+/-standard deviation (SD). The statistical significance was performed by one-way ANOVA for multiple groups followed by Tukey ad hoc statistical test using Graphpad prism 5.0 (La Jolla, CA). In the *in vivo* survival study the number of mice per group was set at n=7-8 mice/group (as specified in the figures). The sample size for each experiment was designed to have 80% power at a two-sided α of 0.05. For the calculation of mouse survival, a Kaplan-Meier Survival Curve statistical analysis was performed in which the uncertainty of the fractional survival of 95% confidence intervals was calculated. Differences between all groups were compared with each other, and were considered significant at values below 0.05.

## SUPPLEMENTARY MATERIALS FIGURES AND TABLES



## References

[1] Voloshin T, Voest EE, Shaked Y (2013). The host immunological response to cancer therapy: an emerging concept in tumor biology. Exp Cell Res.

[2] Shaked Y (2016). Balancing efficacy of and host immune responses to cancer therapy: the yin and yang effects. Nat Rev Clin Oncol.

[3] Shaked Y, Henke E, Roodhart JM, Mancuso P, Langenberg MH, Colleoni M, Daenen LG, Man S, Xu P, Emmenegger U, Tang T, Zhu Z, Witte L (2008). Rapid chemotherapy-induced acute endothelial progenitor cell mobilization: implications for antiangiogenic drugs as chemosensitizing agents. Cancer Cell.

[4] Welford AF, Biziato D, Coffelt SB, Nucera S, Fisher M, Pucci F, Di Serio C, Naldini L, De Palma M, Tozer GM, Lewis CE (2011). TIE2-expressing macrophages limit the therapeutic efficacy of the vascular-disrupting agent combretastatin A4 phosphate in mice. J Clin Invest.

[5] Roodhart JM, Daenen LG, Stigter EC, Prins HJ, Gerrits J, Houthuijzen JM, Gerritsen MG, Schipper HS, Backer MJ, van Amersfoort M, Vermaat JS, Moerer P, Ishihara K (2011). Mesenchymal stem cells induce resistance to chemotherapy through the release of platinum-induced fatty acids. Cancer Cell.

[6] Hasnis E, Alishekevitz D, Gingis-Veltski S, Bril R, Fremder E, Voloshin T, Raviv Z, Karban A, Shaked Y (2014). Anti-Bv8 antibody and metronomic gemcitabine improve pancreatic adenocarcinoma treatment outcome following weekly gemcitabine therapy. Neoplasia.

[7] Beyar-Katz O, Magidey K, Ben-Tsedek N, Alishekevitz D, Timaner M, Miller V, Lindzen M, Yarden Y, Avivi I, Shaked Y (2016). Bortezomib-induced proinflammatory macrophages as a potential factor limiting anti-tumour efficacy. J Pathol.

[8] Gingis-Velitski S, Loven D, Benayoun L, Munster M, Bril R, Voloshin T, Alishekevitz D, Bertolini F, Shaked Y (2011). Host response to short-term, single-agent chemotherapy induces matrix metalloproteinase-9 expression and accelerates metastasis in mice. Cancer Res.

[9] Shaked Y, Ciarrocchi A, Franco M, Lee CR, Man S, Cheung AM, Hicklin DJ, Chaplin D, Foster FS, Benezra R, Kerbel RS (2006). Therapy-induced acute recruitment of circulating endothelial progenitor cells to tumors. Science.

[10] Kim JJ, Tannock IF (2005). Repopulation of cancer cells during therapy: an important cause of treatment failure. Nat Rev Cancer.

[11] Tannock IF (2015). Cancer: resistance through repopulation. Nature.

[12] Klement G, Baruchel S, Rak J, Man S, Clark K, Hicklin D, Bohlen P, Kerbel RS (2000). Continuous low-dose therapy with vinblastine and VEGF receptor-2 antibody induces sustained tumor regression without overt toxicity. J Clin Invest.

[13] Hanahan D, Bergers G, Bergsland E (2000). Less is more, regularly: metronomic dosing of cytotoxic drugs can target tumor angiogenesis in mice. J Clin Invest.

[14] Shaked Y, Emmengger U, Man S, Cervi D, Bertolini F, Ben-David Y, Kerbel RS (2005). The optimal biological dose of metronomic chemotherapy regimens is associated with maximum antiangiogenic activity. Blood.

[15] Shaked Y, Pham E, Hariharan S, Magidey K, Beyar-Katz O, Xu P, Man S, Wu FT, Miller V, Andrews D, Kerbel RS (2016). Evidence implicating immunological host effects in the efficacy of metronomic low-dose chemotherapy. Cancer Res.

[16] Barbolosi D, Ciccolini J, Lacarelle B, Barlesi F, Andre N (2016). Computational oncology—mathematical modelling of drug regimens for precision medicine. Nat Rev Clin Oncol.

[17] Benzekry S, Lamont C, Barbolosi D, Hlatky L, Hahnfeldt P (2017). Mathematical modeling of tumor-tumor distant interactions supports a systemic control of tumor growth. Cancer Res.

[18] Benzekry S, Lamont C, Beheshti A, Tracz A, Ebos JM, Hlatky L, Hahnfeldt P (2014). Classical mathematical models for description and prediction of experimental tumor growth. PLoS Comput Biol.

[19] Benzekry S, Tracz A, Mastri M, Corbelli R, Barbolosi D, Ebos JM (2016). Modeling spontaneous metastasis following surgery: an *in vivo*-in silico approach. Cancer Res.

[20] Ciccolini J, Barbolosi D, Meille C, Lombard A, Serdjebi C, Giacometti S, Padovani L, Pasquier E, Andre N (2017). Pharmacokinetics and pharmacodynamics-based mathematical modeling identifies an optimal protocol for metronomic chemotherapy. Cancer Res.

[21] Benzekry S, Pasquier E, Barbolosi D, Lacarelle B, Barlesi F, Andre N, Ciccolini J (2015). Metronomic reloaded: Theoretical models bringing chemotherapy into the era of precision medicine. Semin Cancer Biol.

[22] Imbs DC, El Cheikh R, Boyer A, Ciccolini J, Mascaux C, Lacarelle B, Barlesi F, Barbolosi D, Benzekry S (2017). Revisiting bevacizumab + cytotoxics scheduling using mathematical modeling: proof of concept study in experimental non-small cell lung carcinoma. CPT Pharmacometrics Syst Pharmacol.

[23] Macheras P, Iliadis A (2016). Modeling in Biopharmaceutics, Pharmacokinetics and Pharmacodynamics.

[24] Innocenti F, Danesi R, Di Paolo A, Agen C, Nardini D, Bocci G, Del Tacca M (1995). Plasma and tissue disposition of paclitaxel (taxol) after intraperitoneal administration in mice. Drug Metab Dispos.

[25] Friedl P, Wolf K (2003). Tumour-cell invasion and migration: diversity and escape mechanisms. Nat Rev Cancer.

[26] Rachman-Tzemah C, Zaffryar-Eilot S, Grossman M, Ribero D, Timaner M, Maki JM, Myllyharju J, Bertolini F, Hershkovitz D, Sagi I, Hasson P, Shaked Y (2017). Blocking surgically induced lysyl oxidase activity reduces the risk of lung metastases. Cell Rep.

[27] Holohan C, Van Schaeybroeck S, Longley DB, Johnston PG (2013). Cancer drug resistance: an evolving paradigm. Nat Rev Cancer.

[28] Katz OB, Shaked Y (2015). Host effects contributing to cancer therapy resistance. Drug Resist Updat.

[29] Shree T, Olson OC, Elie BT, Kester JC, Garfall AL, Simpson K, Bell-McGuinn KM, Zabor EC, Brogi E, Joyce JA (2011). Macrophages and cathepsin proteases blunt chemotherapeutic response in breast cancer. Genes Dev.

[30] Chan TS, Hsu CC, Pai VC, Liao WY, Huang SS, Tan KT, Yen CJ, Hsu SC, Chen WY, Shan YS, Li CR, Lee MT, Jiang KY (2016). Metronomic chemotherapy prevents therapy-induced stromal activation and induction of tumor-initiating cells. J Exp Med.

[31] Shaked Y, Kerbel RS (2007). Antiangiogenic strategies on defense: on the possibility of blocking rebounds by the tumor vasculature after chemotherapy. Cancer Res.

[32] Bertolini F, Paul S, Mancuso P, Monestiroli S, Gobbi A, Shaked Y, Kerbel RS (2003). Maximum tolerable dose and low-dose metronomic chemotherapy have opposite effects on the mobilization and viability of circulating endothelial progenitor cells. Cancer Res.

[33] Hasnis E, Alishekevitz D, Gingis-Veltski S, Bril R, Fremder E, Voloshin T, Raviv Z, Karban A, Shaked Y (2014). Anti-Bv8 antibody and metronomic gemcitabine improve pancreatic adenocarcinoma treatment outcome following weekly gemcitabine therapy. Neoplasia.

[34] Pasquier E, Kavallaris M, Andre N (2010). Metronomic chemotherapy: new rationale for new directions. Nat Rev Clin Onco.

[35] Kareva I (2017). A combination of immune checkpoint inhibition with metronomic chemotherapy as a way of targeting therapy-resistant cancer cells. Int J Mol Sci.

[36] Parra K, Valenzuela P, Lerma N, Gallegos A, Reza LC, Rodriguez G, Emmenegger U, Di Desidero T, Bocci G, Felder MS, Manciu M, Kirken RA, Francia G (2017). Impact of CTLA-4 blockade in conjunction with metronomic chemotherapy on preclinical breast cancer growth. Br J Cancer.

[37] Loven D, Hasnis E, Bertolini F, Shaked Y (2013). Low-dose metronomic chemotherapy: from past experience to new paradigms in the treatment of cancer. Drug Discov Today.

[38] Pasquier J, Thawadi HA, Ghiabi P, Abu-Kaoud N, Maleki M, Guerrouahen BS, Vidal F, Courderc B, Ferron G, Martinez A, Al Sulaiti H, Gupta R, Rafii S (2014). Microparticles mediated cross-talk between tumoral and endothelial cells promote the constitution of a pro-metastatic vascular niche through Arf6 up regulation. Cancer Microenviron.

[39] Zandvliet AS, Schellens JH, Beijnen JH, Huitema AD (2008). Population pharmacokinetics and pharmacodynamics for treatment optimization in clinical oncology. Clin Pharmacokinet.

[40] Meille C, Barbolosi D, Ciccolini J, Freyer G, Iliadis A (2016). Revisiting dosing regimen using pharmacokinetic/pharmacodynamic mathematical modeling: densification and intensification of combination cancer therapy. Clin Pharmacokinet.

[41] Skipper HE, Schabel FM, Wilcox WS (1964). Experimental evaluation of potential anticancer agents. Xiii. On the criteria and kinetics associated with “curability” of experimental leukemia. Cancer Chemother Rep.

[42] Norton L, Simon R (1977). Tumor size, sensitivity to therapy, and design of treatment schedules. Cancer Treat Rep.

[43] Simeoni M, Magni P, Cammia C, De Nicolao G, Croci V, Pesenti E, Germani M, Poggesi I, Rocchetti M (2004). Predictive pharmacokinetic-pharmacodynamic modeling of tumor growth kinetics in xenograft models after administration of anticancer agents. Cancer Res.

[44] Simeoni M, De Nicolao G, Magni P, Rocchetti M, Poggesi I (2013). Modeling of human tumor xenografts and dose rationale in oncology. Drug Discov Today Technol.

[45] Faivre C, El Cheikh R, Barbolosi D, Barlesi F (2017). Mathematical optimisation of the cisplatin plus etoposide combination for managing extensive-stage small-cell lung cancer patients. Br J Cancer.

[46] Benzekry S, Hahnfeldt P (2013). Maximum tolerated dose versus metronomic scheduling in the treatment of metastatic cancers. J Theor Biol.

[47] Karagiannis GS, Pastoriza JM, Wang Y, Harney AS, Entenberg D, Pignatelli J, Sharma VP, Xue EA, Cheng E, D’Alfonso TM, Jones JG, Anampa J, Rohan TE (2017). Neoadjuvant chemotherapy induces breast cancer metastasis through a TMEM-mediated mechanism. Sci Transl Med.

[48] Kioi M, Vogel H, Schultz G, Hoffman RM, Harsh GR, Brown JM (2010). Inhibition of vasculogenesis, but not angiogenesis, prevents the recurrence of glioblastoma after irradiation in mice. J ClinInvest.

[49] Ahn GO, Tseng D, Liao CH, Dorie MJ, Czechowicz A, Brown JM (2010). Inhibition of Mac-1 (CD11b/CD18) enhances tumor response to radiation by reducing myeloid cell recruitment. Proc Natl Acad Sci U S A.

[50] Timaner M, Bril R, Kaidar-Person O, Rachman-Tzemah C, Alishekevitz D, Kotsofruk R, Miller V, Nevelsky A, Daniel S, Raviv Z, Rotenberg SA, Shaked Y (2015). Dequalinium blocks macrophage-induced metastasis following local radiation. Oncotarget.

[51] Bono A, Bianchi P, Locatelli A, Calleri A, Quarna J, Antoniott P, Rabascio C, Mancuso P, Andreoni B, Bertolini F (2010). Angiogenic cells, macroparticles and RNA transcripts in laparoscopic vs open surgery for colorectal cancer. Cancer Biol Ther.

[52] Leonard R, Hennessy BT, Blum JL, O’Shaughnessy J (2011). Dose-adjusting capecitabine minimizes adverse effects while maintaining efficacy: a retrospective review of capecitabine for metastatic breast cancer. Clin Breast Cancer.

[53] Evans WE, Relling MV, Rodman JH, Crom WR, Boyett JM, Pui CH (1998). Conventional compared with individualized chemotherapy for childhood acute lymphoblastic leukemia. N Engl J Med.

[54] Bocci G, Kerbel RS (2016). Pharmacokinetics of metronomic chemotherapy: a neglected but crucial aspect. Nat Rev Clin Oncol.

[55] Minasian L, Rosen O, Auclair D, Rahman A, Pazdur R, Schilsky RL (2014). Optimizing dosing of oncology drugs. Clin Pharmacol Ther.

[56] Shaked Y, Tang T, Woloszynek J, Daenen LG, Man S, Xu P, Cai SR, Arbeit JM, Voest EE, Chaplin DJ, Smythe J, Harris A, Nathan P (2009). Contribution of granulocyte colony-stimulating factor to the acute mobilization of endothelial precursor cells by vascular disrupting agents. Cancer Res.

[57] Egeblad M, Werb Z (2002). New functions for the matrix metalloproteinases in cancer progression. Nat Rev Cancer.

[58] Shaked Y, Bertolini F, Man S, Rogers MS, Cervi D, Foutz T, Rawn K, Voskas D, Dumont DJ, Ben-David Y, Lawler J, Henkin J, Huber J (2005). Genetic heterogeneity of the vasculogenic phenotype parallels angiogenesis: implications for cellular surrogate marker analysis of antiangiogenesis. Cancer Cell.

[59] Philpott NJ, Turner AJ, Scopes J, Westby M, Marsh JC, Gordon-Smith EC, Dalgleish AG, Gibson FM (1996). The use of 7-amino actinomycin D in identifying apoptosis: simplicity of use and broad spectrum of application compared with other techniques. Blood.

[60] Burnham KP, Anderson DR (2003). Model selection and multimodel inference: a practical information-theoretic approach.

[61] Hoel PG (1962). Introduction to mathematical statistics.

