# Supplementary material for: Dose- and time-dependence of the host-mediated response to paclitaxel therapy: a mathematical modeling approach
Source: Oncotarget. 2017 Dec 20;9(2):2574–90. doi: 10.18632/oncotarget.23514 (PMC5788661; doi:10.18632/oncotarget.23514)
Supplement: Supplementary file 1 [file oncotarget-09-2574-s001.pdf]

# Dose- and time-dependence of the host-mediated response to paclitaxel therapy: a mathematical modeling approach

## SUPPLEMENTARY MATERIALS

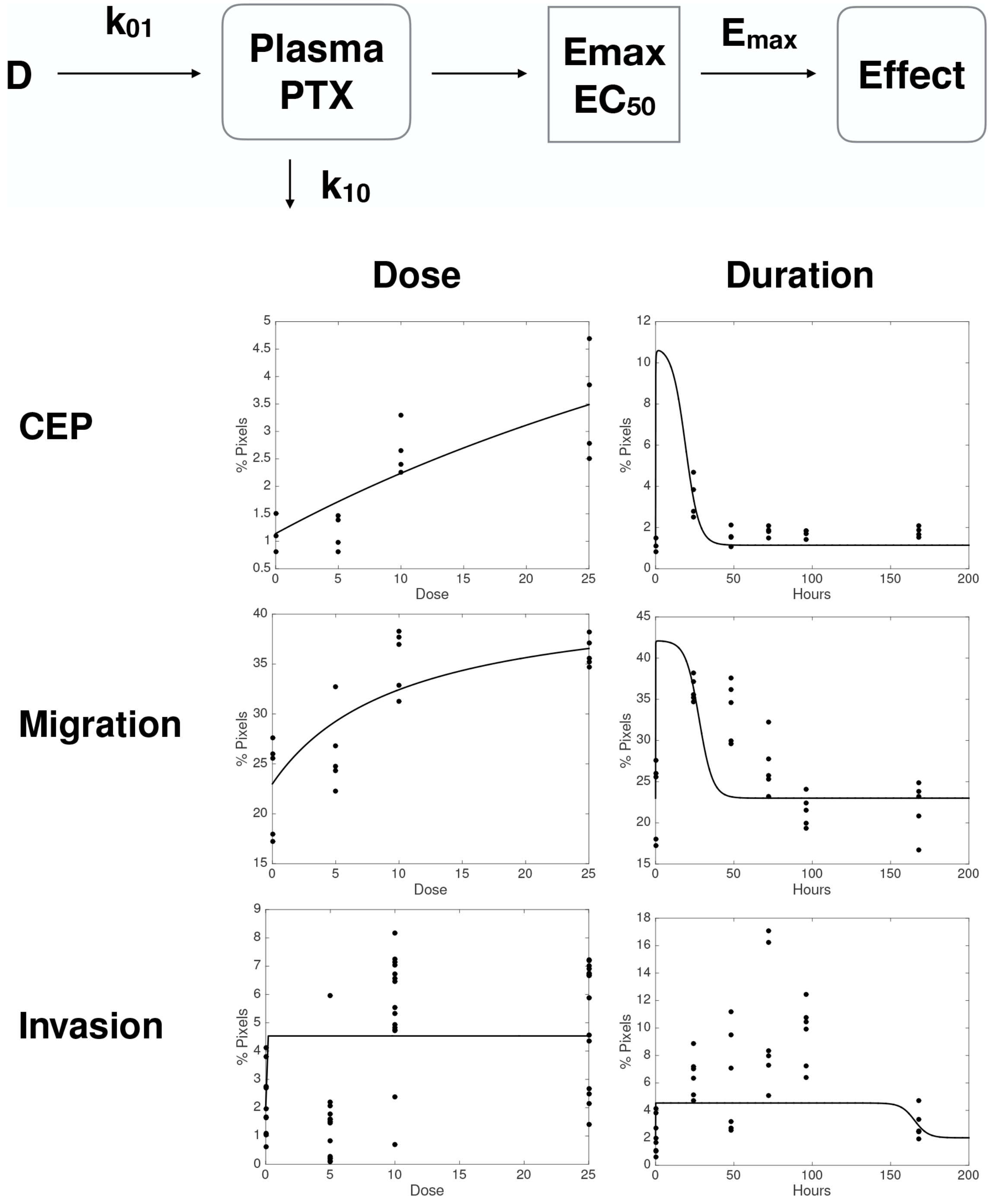


**Supplementary Figure 1: Fits of the one-compartment PK model.** Fits of models employing linking the effect directly to the plasma concentration of paclitaxel, modeled with one-compartmental pharmacokinetics with adsorption (1). Independent models for each of the measured variable (i.e., different values of the model's parameters for CEP, migration and invasion).

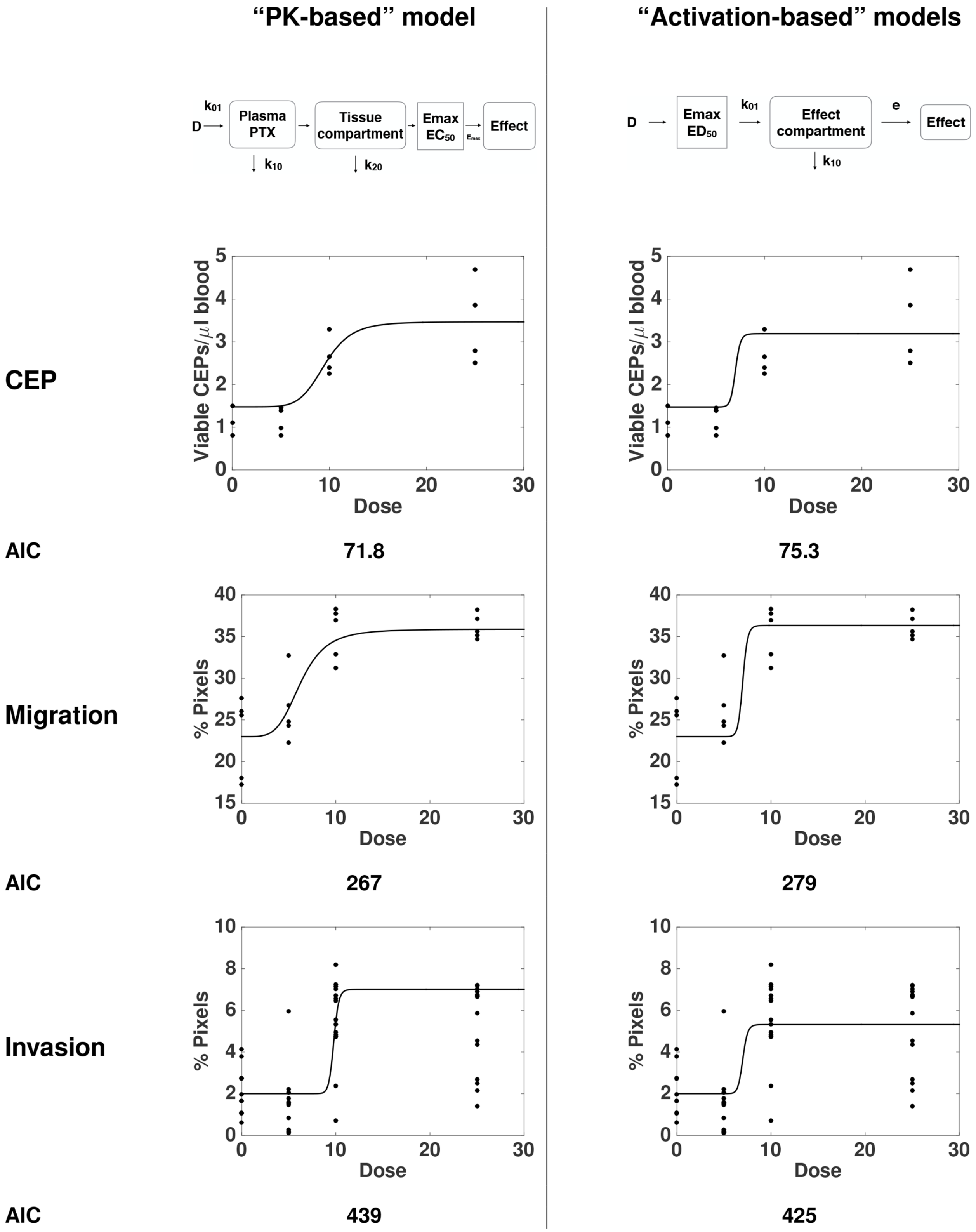


**Supplementary Figure 2: PK-based versus activation-based models: dose-dependence.** Dose-dependence corresponding to the fits reported in Figure 3.

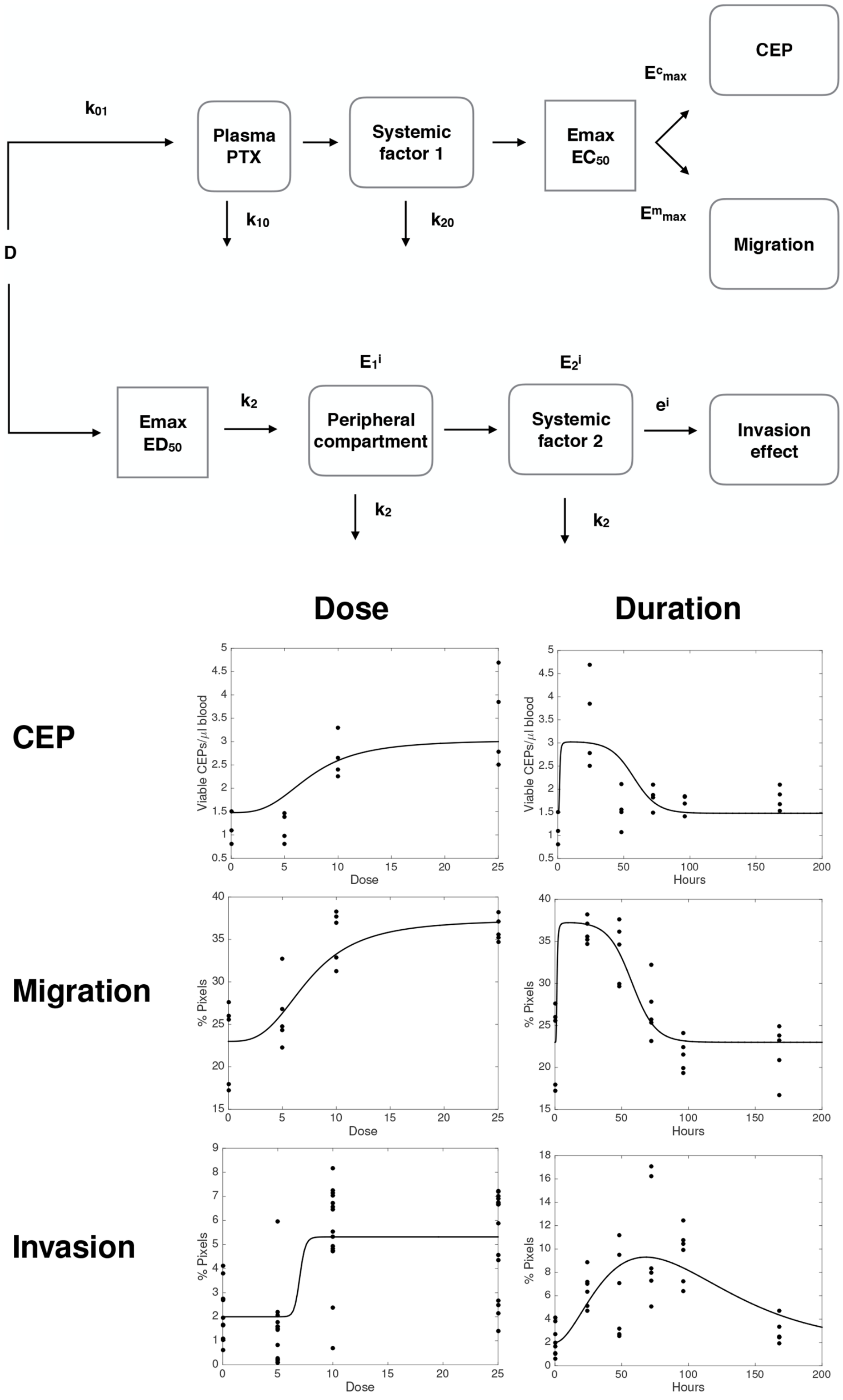


**Supplementary Figure 3: Best-fit of model 1 (same $EC_{50}$ and $k_{20}$).** Fit resulting from likelihood maximization of a model where $EC_{50}$ and $k_{20}$ are set to be the same for the CEP and migration variables (model 1).

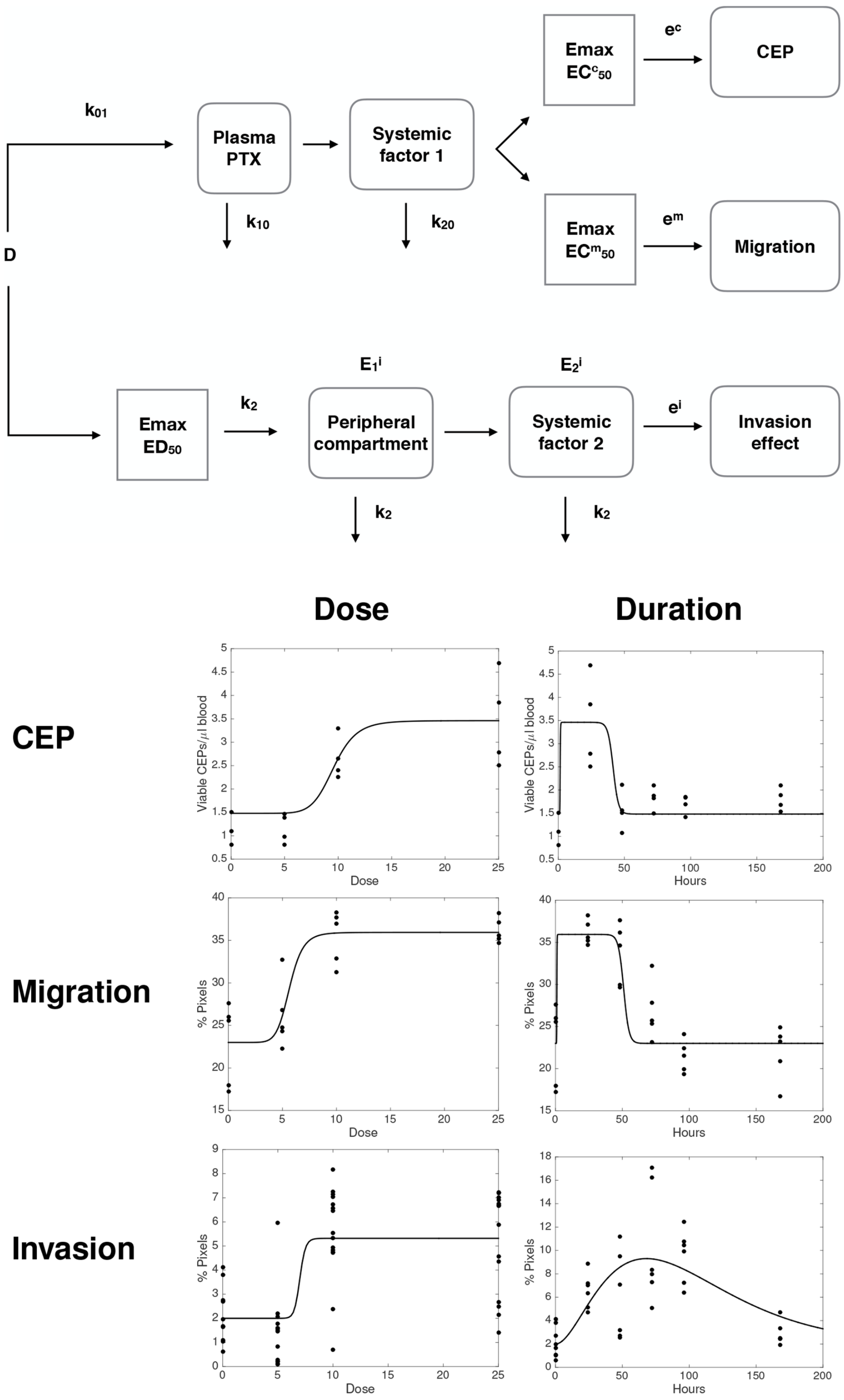


**Supplementary Figure 4: Best-fit of model 2 (same $k_{20}$ but distinct $EC_{50}$).** Fit resulting from likelihood maximization of a model where $k_{20}$ is set to be the same for the CEP and migration variables but $EC_{50}$ considered distinct (model 2).

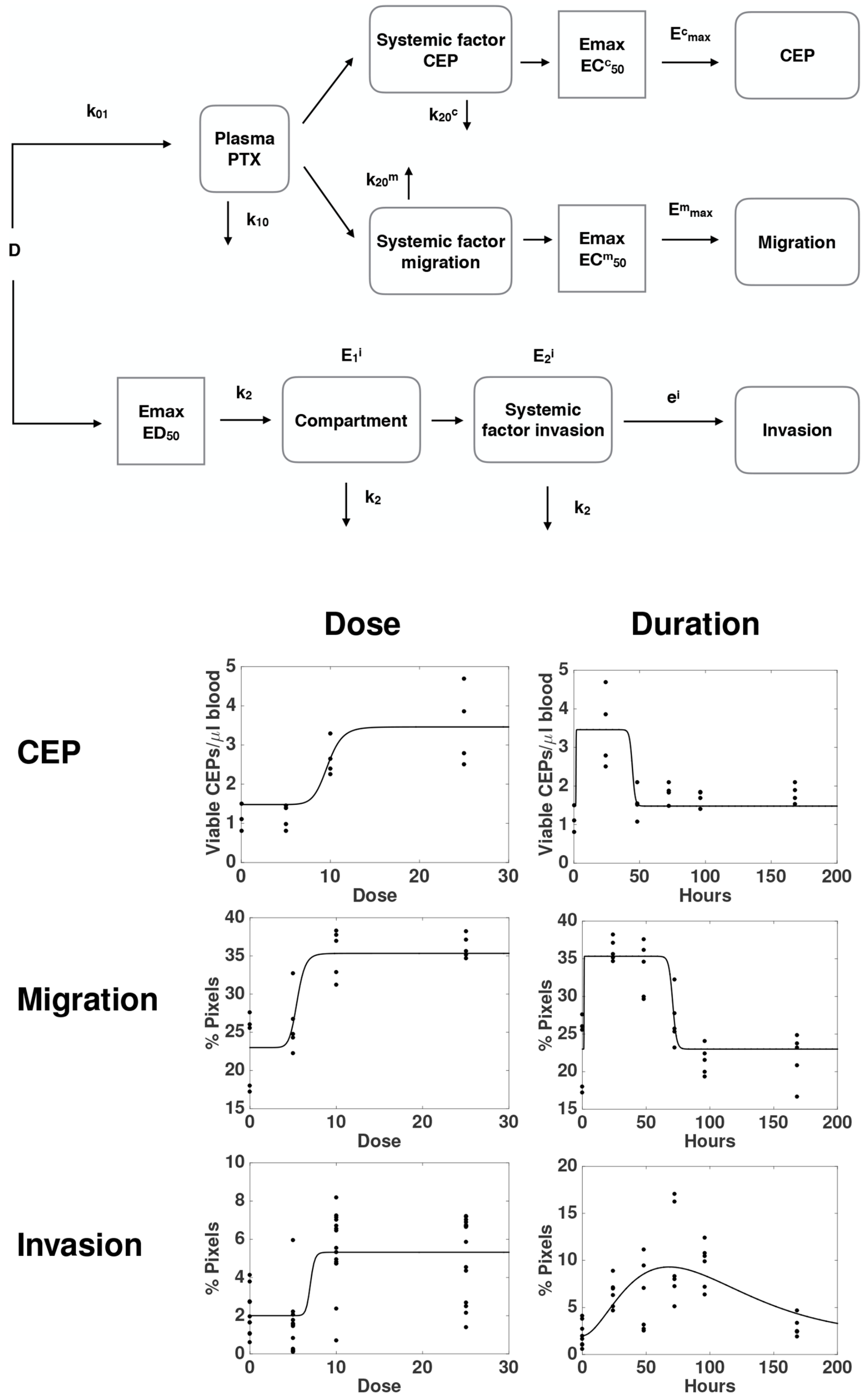


**Supplementary Figure 5: Best-fit of model 4 (distinct $EC_{50}$ and $k_{20}$).** Fit resulting from likelihood maximization of a model where $EC_{50}$ and $k_{20}$ are set to be different for the CEP and migration variables (model 4).

**Supplementary Table 1: p-values of two by two likelihood-ratio tests among all the global models**

| | Model 3 | Model 2 | Model 4 |
|---|---|---|---|
| **Model 1** | <0.0001 | <0.0001 | <0.0001 |
| **Model 3** | - | - | 0.777 |
| **Model 2** | - | - | 0.024 |

Likelihood-ratio tests were performed for nested models to determine significance of the fit improvement from a model with more parameters. Such tests for Model X versus Model Y are only defined when Model X has more parameters than Model Y.

**Supplementary Table 2: Parameter values from the likelihood maximization of model 3**

| Model | Par. | Unit | Value | Status |
|---|---|---|---|---|
| Model 3 | $ED_{50}$ | mg· kg−1 | 7 | fixed |
| | $\gamma_D$ | - | 28 | fixed |
| | $EC_{50}$ | mg· kg$^{-1}$ ·ml$^{-1}$ | 0.726 | fit |
| | $\gamma_C$ | - | 7.91 | fit |
| | $V_d$ | ml | 18.5 | fixed |
| | $k_{01}$ | hour$^{-1}$ | 0.99 | fixed |
| | $k_{10}$ | hour$^{-1}$ | 0.231 | fixed |
| | $k_{20,mig}$ | hour$^{-1}$ | 0.033 | fit |
| | $k_{20,CEP}$ | hour$^{-1}$ | 0.0615 | fit |
| | $k_2$ | hour$^{-1}$ | 0.0294 | fit |
| | $\varepsilon_{inv}$ | % pixels | 2 | fixed |
| | $\varepsilon_{mig}$ | % pixels | 23 | fixed |
| | $\varepsilon_{CEP}$ | Viable CEPs/µl blood | 1.48 | fixed |
| | $e_{inv}$ | % pixels | 0.794 | fit |
| | $E_{max,mig}$ | % pixels | 35.4 | fit |
| | $E_{max,CEP}$ | Viable CEPs/µl blood | 3.46 | fit |

Parameters of the final model were either fitted to the data using likelihood maximization (fit), or fixed either directly from the data or the literature. Paclitaxel pharmacokinetics parameters $V_D$, $k_{01}$ and $k_{10}$ were retrieved from (1). Parameters $\varepsilon_{inv}$, $\varepsilon_{mig}$ and $\varepsilon_{CEP}$ for the baseline values of the measured effects were taken from the control data. The dose threshold for activation of the host response on invasion, $ED_{50}$ was arbitrarily set to 7 mg/kg from the observations of the dose-response curve showing a threshold between 5 and 10 mg/kg (Figure 1). The associated slope of the $E_{max}$ function, $\gamma_D$ was fixed from preliminary fits to the dose-dependence of the effect on invasion. All the other parameters were obtained by fitting the model to the data.
